# Study on the nonfatigue and fatigue states of orchard workers based on electrocardiogram signal analysis

**DOI:** 10.1038/s41598-022-08705-z

**Published:** 2022-03-22

**Authors:** Ruitao Gao, Huachao Yan, Jieli Duan, Yu Gao, Can Cao, Lanxiao Li, Liang Guo

**Affiliations:** 1 College of Engineering, South China Agricultural University, Wushan Road, Tianhe District, Guangzhou, 510642 China; 2 Guangdong Laboratory for Lingnan Modern Agriculture, Wushan Road, Tianhe District, Guangzhou, 510642 China

**Keywords:** Health occupations, Bioinformatics, Electrophysiology

## Abstract

In recent years, fatigue has become an important issue in modern life that cannot be ignored, especially in some special occupations. Agricultural workers are high-risk occupations that, under fatigue conditions over a long period, will cause health problems. In China, since very few studies have focused on the fatigue state of agricultural workers, we were interested in using electrocardiogram (ECG) signals to analyze the fatigue state of agricultural workers. Healthy agricultural workers were randomly recruited from hilly orchards in South China. Through the field experiment, 130 groups of 5-min interval ECG signals were collected, and we analyzed the ECG signal by HRV. The time domain (meanHR, meanRR, SDNN, RMSSD, SDSD, PNN20, PNN50 and CV), frequency domain (VLF percent, LF percent, HF percent, LF norm, HF norm and LF/HF) and nonlinear parameters (SD1, SD2, SD1/SD2 and sample entropy) were calculated and Spearman correlation coefficient analysis and Mann–Whitney U tests were performed on each parameter for further analysis. For all subjects, nine parameters were slightly correlated in nonfatigue and fatigue state. Six parameters were significantly increased and ten HRV parameters were significantly decreased compared the nonfatigue state. As for males, fifteen parameters were significantly different, and for females, eighteen parameters were significantly different. In addition, the probability density functions of SDNN, SDSD, VLF%, HFnorm and LF/HF were significantly different in nonfatigue and fatigue state for different genders, and the nonlinear parameters become more discrete compared the nonfatigue state. Finally, we obtained the most suitable parameters, which reflect the fatigue characteristics of orchard workers under different genders. The results have instructional significance for identifying fatigue in orchard workers and provide a convincing and valid reference for clinical diagnosis.

## Introduction

Fatigue is a common issue affecting people from all walks of life. The risk of accidents will significantly increase when people are under fatigue. Fatigue-related disorders, such as cardiovascular disease, are a major topic in occupation and public health^1^. It is generally believed that fatigue is mainly due to a large consumption of physical and mental capability, which is shown as a decline in the function of human organs or cells and a reduction in reaction ability^2^. In the field of occupational safety and health, fatigue could be defined as the mental or physical decline of people in work with long duration and high labor intensity. From the perspective of physiology, fatigue could also be understood as a method of self-protection for relieving functional consumption. At the same time, fatigue is also a subjective feeling. Under the influence of many factors, the measurement of fatigue could not be studied by simply linear calculation of working intensity.

In many industries, massive research work has been done to measure fatigue levels. Huang et al.^3^ analyzed the applicability of “Subjective Fatigue Symptoms” revised by the Japan Institute of Industrial Hygiene in the Chinese Manufacturing Industry. Binoosh et al.^4^ designed the assembly test of workers and used several fatigue questionnaires to predict the fatigue degree of workers. The results show that the Borg CR10 scale has better performance than the Samn-Perelli fatigue scale (SPFS) in fatigue predictability. Research on driving fatigue is currently a hot topic. A series of studies have been performed to recognize driving fatigue from the perspective of physiological signals. These signals include electroencephalogram (EEG)^5,6^, surface electromyography (sEMG)^7,8^ and electrocardiogram (ECG)^9^, and researchers have also made rich achievements regarding driving fatigue. Similarly, miners' fatigue and behavior safety have gradually attracted extensive attention. Tian et al. ^10^ revealed the relationship between miners’ fatigue and unsafe behavior by recording physiological behavior with eye trackers, behavior recorders and arm adjustment testers. Risk assessments of occupational diseases, such as anthraciosis pneumoconiosis and lung cancer, have also been studied^11,12^. In the construction industry, the fatigue of construction workers was usually scaled by subjective questionnaires in most studies. Qi^13^ and Lou^14^ used a questionnaire to study the relationship between workers’ fatigue and construction quality, and a structural equation model was applied to fit the fatigue level and obtained good results. Dong et al.^15^ studied the influence of two fatigue factors on unsafe behavior in construction workers: overtime and irregular working arrangement. The results showed that two fatigue factors were harmful to safety for workers. Xiang et al.^16^. designed a fatigue test of construction workers based on the handing error rate and discussed the relationship between a variety of physiological parameters and unsafe behavior. The study found that the number of errors was positively correlated with electrodermal activity (EDA), respiration (RESP) and LF/HF and negatively correlated with the standard deviation of EDA, skin temperature (SKT), R-R interval, SDNN and HFnorm.

Agriculture is one of the most hazardous productive sectors around the world^17^, and studies have shown that the quality of life of farmers is closely related to fatigue^18^. In addition, farmers experiencing long-term fatigue have an increased risk of hypertension, heart diseases and suicide^19–21^. Orchard workers are a population at risk for serious occupational injuries and illnesses. In China, research on the occupational health and safety of orchard works is quite rare, while developed countries have pursued research on this issue^22,23^. Moreover, there are many studies on the optimization design of orchard production equipment to reduce the risk of unsafe behavior of orchard workers^24–26^.

Based on the above studies, many scholars have studied the fatigue of workers in the construction industry and the mining industry. However, in agriculture, the recognition of farmers’ fatigue has received less attention, and the occupational health problems of farmers need to be invested more. With the development of hardware technology, wireless biosensors are rapidly growing in research in psychology, medicine and ergonomics, and these sensors can collect sufficient and continuous physiological information in real time and transmit it via wireless or Bluetooth. Among the kinds of physiological signals, ECG signals are widely used in the detection of comprehensive fatigue in many fields, and many studies have described the connection between cardiac rhythm and the autonomic nervous system (ANS)^27,28^. In a case study, we will combine the questionnaire survey and physiological measurement to analyze fatigue orchard workers. The purpose of this study was to provide effective parameters for fatigue in orchard workers, and the results can be used for fatigue prediction and referred to clinically for occupational disease diagnosis.

## Method

### Ethic statement

The whole experiment was approved by the Industrial Design Ethics Committee of South China Agricultural University and followed the 1964 Declaration of Helsinki. All subjects with informed consent and submitted written consent after verbal explanation of the study. The experiments and the fatigue questionnaire were completed in voluntary and anonymously All experiments were performed in accordance with test criteria and regulations of Industrial Design Department of College of Engineering.

### Participants

The method of stratified random sampling was adopted for the selection of subjects. We selected 65 workers from the hilly orchard in Guangzhou and South China Agricultural University, the typical hilly orchard in South China, as subjects, and raw data were obtained through field collection. The basic information of subjects is as follows: the average age was 30.7 ± 5.1, the average weight was 68.79 ± 3.4 kg, the subjects got sufficient sleep the day before the experiment and no alcohol or caffeine intake within 24 h. All subjects had no history of heart disease and were in good health. Strenuous exercise was avoided, and food intake was limited to 1 h before the experiment.

### Experimental procedure

The experiment of the study was designed to obtain the ECG signal of orchard workers in fatigue and nonfatigue states. Individual information, such as age, weight and height, was recorded in preparation. A set of questionnaires (FS-14) was given to subjects to complete to measure comprehensive fatigue before and after work in the field study. After daily work, most subjects should experience fatigue. The ECG data were collected in the nonfatigue state and fatigue state. In this way, the ECG signals of orchard workers under fatigue and nonfatigue states were obtained.

**Figure 1:**
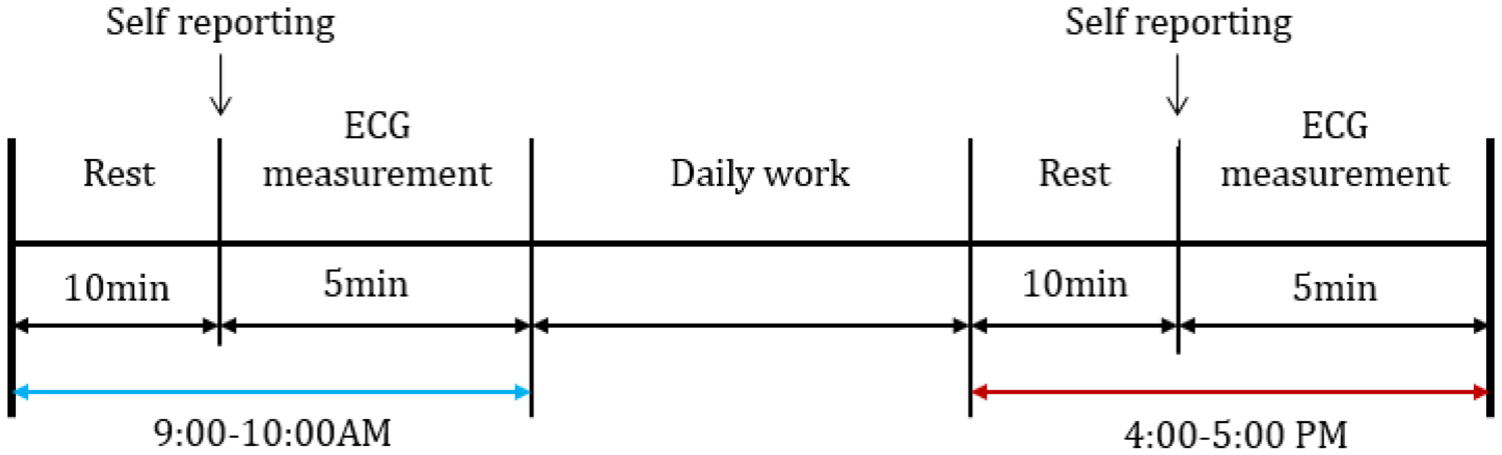
The flow chart of total experiment.

First, all subjects rested for 10 min and then completed the questionnaire to report the current fatigue state. After that, the ECG signal was collected to obtain the baseline state of orchard workers. Next. The workers were required to carry out field activities as usual. After the end of the working day, each subject was required to follow a 10-min rest, and the questionnaire needed to be completed again. Finally, 5 min of ECG signal acquisition was performed to record the fatigue state of the orchard workers after finishing a day’s task. Figure 1 shows the test flow.

**Figure 2:**
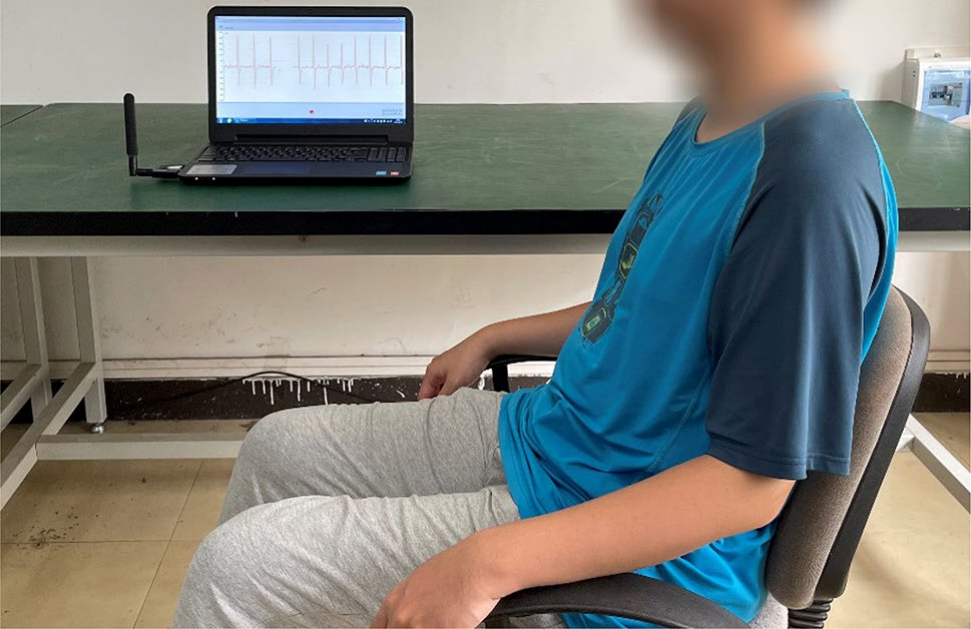
Real-time ECG signal acquisition of orchard workers.

The specific details in the test are as follows:

1. This experiment considered the factors that affect fatigue caused by circadian fluctuations^29,30^. Therefore, to avoid this factor in ECG signal collection, the experiments were completed in the same time session (9:00–10:00 a.m. and 4:00–5:00 p.m.).
2. The subjective questionnaire of the Fatigue Scale proposed by Chalder et al.^31^ was to measure comprehensive fatigue state in this study, it contains 14 items which include 8 physical fatigue questions and 6 mental questions, and the fatigue scale is a 5-point Likert scales from 1 = “strongly disagree” to “strong agree” applied to all items.
3. During the 10-min rest and more than 5-min ECG signal collection, all subjects needed to remain in a relaxed upright posture. Upright posture means required subjects to put hand on thighs with elbow angle at 110°–135°and maintain seating posture with knee angle and thigh-angle at 105–130° and 100–125°, respectively.
4. The ECG signal was measured by the method of three-lead chest association. After wiping the skin with alcohol, three electrodes were placed on the chest of subjects. The positions of the three electrodes were as follows: the positive electrode was affixed to the sternum and the 3rd costa, the negative electrode was affixed to the left margin of the sternum and the 5th costa, and the reference electrode was affixed to 1 cm below the xiphoid process. After collecting the ECG signal, ECG characteristics can be obtained by filtering and denoising^32^. The detection method proposed by Wu^33^ was used to extract the R peak value. The real-time acquisition of the ECG signal is shown in Fig. 2.

### Experimental condition

This study aimed to analyze the ECG signal and identify fatigue characteristics of orchard workers. The measurement conditions were as follows: the temperature was 27.8–33.5 °C, the altitude was 500–720 m, the wetness was 71.7–84.3%, and the atmospheric pressure was 998.6–1002.3 hPa. Litchi, longan and citrus are the main crops in this hilly orchard.

### Experimental equipment

In this study, Ag/AgCl electrodes were selected as the skin surface electrode for ECG acquisition because of their better conductivity and stability. The ErgoLAB man–machine synchronization system developed by Jinfa Technology Co., Ltd. was able to collect multiple physiological signals at the same time, such as sEMG, EOG, ECG and RESP.

**Figure 3:**
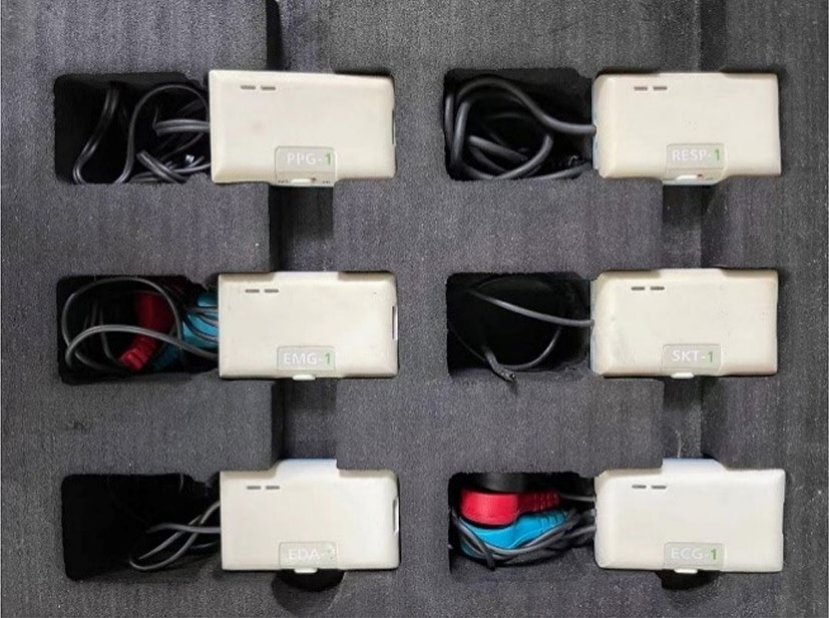
Physiological sensors.

Physiological sensors matched with the Man machine system system are shown in Fig. 3. In particular, the sampling frequency of the ECG sensor is 256 Hz, and the communication mode is 2.4 GHz two-way digital wireless transmitter. Pure ECG signals can be obtained by noise reduction and filtering.

### Computational method

**Figure 4:**
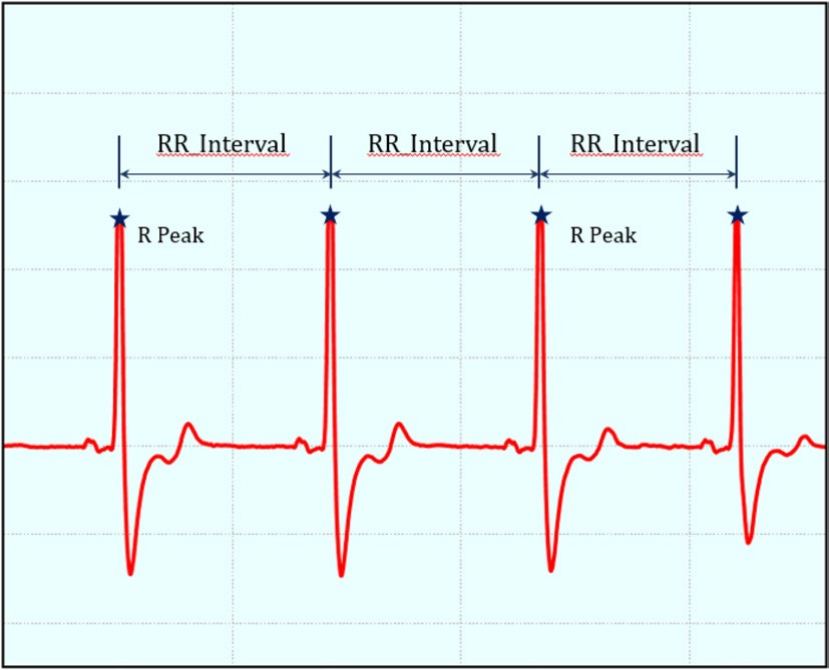
Real time ECG signal.

The ECG signal (Fig. 4) is the biological reaction on the body surface in the process of heart activity electrical signals generated. Heart rate variability (HRV) is the classical method for analyzing ECG signals and refers to the variability of small differences between adjacent heartbeat cycles. It is generally accepted that HRV describes the adaptive changes of autonomic nervous activity (ANS) in response to unpredictable factors, such as cardiac disease, stress, fatigue and drowsiness^33^. The sympathetic nervous system (SNS) and parasympathetic nervous system (PNS) are components of the ANS, and the balancing action of the SNS and PNS branches of the ANS controls heart rhythm. HRV can quantitatively assess the tension and balance of SNS and PNS activity and its effect on cardiovascular system activity under the fatigue state, and reflect the condition of fatigue from physical and mental load. The most conspicuous feature of HRV is the abnormal sensitivity which is contains subtle information about cardiovascular regulation. Many application sceneries where HRV have been found available include evaluation of physical training intensity and clinical diagnosis of cardiac illness.

In this study, we concentrated on the study of orchard workers under fatigue and nonfatigue states in HRV and summarized the changing characteristics of physiological parameters. HRV analysis mainly includes time domain analysis, frequency domain analysis and nonlinear analysis.

#### Time domain analysis

Time domain analysis is a method for calculating the differences in the R-R interval by discrete statistical analysis. The RR interval is the time difference between two between the R peaks of two consecutive heart beats in the ECG, In this section, we calculated the key time domain parameters of HRV. The calculation methods of each parameter are as follows:

1. Mean RR Interval (meanRR): The mean RR interval can be calculated using the following formula:
  1 $$meanRR=\frac{1}{N}\sum_{i=1}^{N}R{R}_{i}$$
2. Mean Heart Rate (meanHR): The mean Heart Rate is defined as:
  2 $$meanHR=\frac{60}{meanRR}$$
3. SDNN is the standard deviation of all RR intervals over the whole collection time, which reflects the comprehensive change in heart rate variation in this period. The formula for calculation of SDNN is as follows:
  3 $$SDNN=\sqrt{\frac{1}{N}\sum_{i=1}^{N}{(R{R}_{i}-meanRR)}^{2}}$$
4. The root mean square of successive differences (RMSSD) between the R-R interval is a key time domain parameter to estimate vagus nerve changes, which also reflects the high-frequency component in HRV. The RMSSD is defined as:
  4 $$RMSSD=\sqrt{\frac{1}{N-1}\sum_{i-1}^{N-1}{(R{R}_{i+1}-R{R}_{i})}^{2}}$$
5. SDSD is the standard deviation of the difference between adjacent intervals, which can be calculated in the following formula:
  5 $$SDSD=\sqrt{\frac{1}{N}\sum_{i=1}^{N}{[\left({RR}_{i}-{RR}_{i+1}\right)-(meanRR-R{R}_{i+1})]}^{2}}$$
6. The coefficient of variation (CV) is the ratio of the SDNN to meanHR, and the formula is defined as follows:
  6 $$CV=\frac{SDNN}{meanRR}\times 100\%$$
7. PNN20 is the proportion of the difference between adjacent intervals of more than 20 ms in a period of time. PNN20 is defined as follows:
  7 $$PNN20=\frac{NN20}{N-1}\times 100\%$$
  where NN20 is the number of successive R-R interval pairs that differ by more than 20 ms.
8. PNN50 is the proportion of the difference between adjacent intervals of more than 50 ms in a period of time. The formula for calculation of PNN50 is as follows:
  8 $$PNN50=\frac{NN50}{N-1}\times 100\%$$

#### Frequency domain analysis

The frequency domain analysis of HRV analyzes the characteristics of heart rate from the power spectrum. There is a certain correlation between frequency domain analysis and time domain analysis, but frequency domain analysis can reveal more complex heart rate changes. The classical spectrum estimation of fast Fourier transformation (FFT) and the modern spectrum estimation method of the autoregressive (AR) model are always used to obtain the power spectrum with frequency as the X-axis and power amplitude as the Y-axis^34^. The power distribution of the ECG signal in different frequency bands can be characterized quantitatively by spectral analysis. The frequency domain analysis of the ECG signal provides four components, including ultra low frequency (ULF, ≤ 0.003 Hz), very low frequency (VLF, 0.003–0.04 Hz), low frequency (LF, 0.04–0.15 Hz) and high frequency (HF, 0.15–0.4 Hz). These components were generally expressed in normalized units, which represent the dominance of ANS, SNS and PNS activities for cardiac rhythm^35,36^. Therefore, we extracted the relative powers (%) of VLF, LF and HF and normalized the powers of LF and HF and the ratio of LF and HF. Since ULF requires a long recording period of least 24 h while the production mechanisms of ULF band is still in dispute so the characteristic parameters of ULF are not calculated in this study. The formula for the calculation of mentioned frequency domain component is as follows:

9 $$VL{F}_{percent}=\frac{VLF \; power}{Total \; power}\times 100\%$$

10 $$L{F}_{percent}=\frac{LF \; power}{Total \; power}\times 100\%$$

11 $$H{F}_{percent}=\frac{HF \; power}{Total \; power}\times 100\%$$

12 $$L{F}_{norm}=\frac{LF\; power}{Total \;power-uLF \;power}$$

13 $$H{F}_{norm}=\frac{HF \;power}{Total \;power-uLF\; power}$$

#### Nonlinear analysis

Nonlinear means that a relationship between parameters cannot be plotted as a straight line^37^. A Poincare plot is a graphed method applied to HRV for nonlinear analysis, which is used to analyze the dispersion of the RR interval and can directly reflect the pattern of every RR interval. The coordinates of the data points in the Poincare plot are determined by the sequence of RR intervals. The data points are plotted with the RR_i_ interval as the abscissa and RR_i+1_ interval as the ordinate. Ellipses are used to fit data points in a Poincare plot, and the shape of the ellipses is determined by the standard deviation of the data point in the T direction and L direction, which are denoted by SD1 and SD2, respectively. Concretely, SD1 specifies the width of the fitting ellipse, and SD2 specifies the length of the fitting ellipse. By calculating the standard deviations of distances of the data point to the line y = x and y = − x + 2meanRR to analyze the dispersion of data points. In short term measurement of HRV, Brennan et al.^38^ proved that SD1 and SD2 are related to the time domain parameters, which can be expressed as formulas (14) and (15). In particular, SD1 indicates the short-term variability caused by respiratory sinus arrhythmia (RSA), and SD2 could measure both the long-term and short-term variability of HRV.

14 $$SD1=\sqrt{\frac{1}{N-1}\sum_{i=1}^{N-1}\frac{{({RR}_{i}-{RR}_{i+1})}^{2}}{2}}=\frac{\sqrt{2}}{2}SDSD$$

15 $$SD2=\sqrt{\frac{1}{N-1}\sum_{i=1}^{N-1}\frac{{({RR}_{i}+R{R}_{i+1}-2meanHR)}^{2}}{2}}=\sqrt{2{SDNN}^{2}-\frac{1}{2}{SDSD}^{2}}$$

where N is the number of successive R–R interval pairs and SDSD and SDNN are shown in Eqs. (3) and (5).

By observing the distribution of data points in the Poincare plot, the abnormal RR interval caused by fatigue can be located. The intuitive difference in the RR interval of orchard workers between fatigue and nonfatigue states was analyzed by Poincaré plots. Therefore, SD1, SD2 and SD1/SD2 were selected to analyze the nonlinear variability of HRV.

#### Sample entropy

Entropy is a measurement of physical properties and was initially defined as the quotient of an infinitesimal amount of heat to the instantaneous temperature. The complexity unpredictability of a sequence signal is also reflected by entropy in informatics, and the principle of entropy is to estimate the complexity by detecting the generation probability of new subsequences in the time domain signal. The approximate entropy and sample entropy are generally used for nonlinear analysis of ECG signals^39–41^. Sample entropy was proposed by Richman et al.^40^, which is also an improvement of approximate entropy. Compared with approximate entropy, the calculation of sample entropy maintains better relative consistency for data with large amplitude, and self-matches are not included in calculating the probability. This means that sample entropy represents more self-similarity in the time series. Therefore, in this study, we selected sample entropy to scale the complexity of the HRV signal. Sample entropy can be obtained by follows:

First, the time series are reconstructed in phase space, and it is assumed that for a group of measured time series ${{\{x}_{n}\}}_{n=1}^{N}$ with a length of N, the reconstructed phase space is defined as Eq. (16):

16 $${X}_{n}=\left\{{x}_{n},{x}_{n+\uptau },\ldots ,{x}_{n+(m-1)\uptau }\right\}\in {R}^{m},n={N}_{0},{N}_{0}+1,\ldots ,N$$

$${N}_{0}=\left(m-1\right)\uptau +1$$

where m is the dimension and τ is the delay time interval of time series ${{\{x}_{n}\}}_{n=1}^{N}$. The distance between components $X(i)$ and $X(j)$ is defined as the maximum distance, which can be expressed as Eq. (17):

17 $$d\left[X\left(i\right),X\left(j\right)\right]=max||x\left(i+k-1\right)-x(j+k-1)||$$

where $k=\mathrm{1,2},\ldots m$ and $i\le N-m+1$. With the center of $y\left(i\right)$ and allowable deviation $r$ in the $m$ dimension space, the probability of the distance between $X\left(i\right)$ and $X\left(j\right)$ of remaining vectors less than $r$ is defined as:

18 $${C}_{i}^{m}(r)=\frac{count(X(j)|d[X\left(i\right),X(j)]\le r)}{N-m} \forall k\ne j$$

where $r$ is a preselected parameter and is empirically taken as $0.1*STD-0.25*STD$. $SDT$ represents the standard deviation of the time series. We selected $r=0.2*STD$ in this study. ${C}_{i}^{m}\left(r\right)$ reflects the degree of correlation between both $X\left(i\right)$ and $X\left(j\right)$, which means the regularity degree of vector $\{X\left(i\right)\}$. The average regularity can be calculated as follows:

19 $${\Phi }^{m}(r)=\frac{\sum_{i=1}^{N-m}{C}_{i}^{m}(r)}{N-m}$$

Through the calculation method above, ${\Phi }^{m+1}(r)$ can be obtained, and the sample entropy can be expressed as:

20 $$SampleEn(m,r,N)=\underset{N\to \infty }{\mathrm{lim}}\left\{{\mathrm{ln}}\frac{{\Phi }^{m}(r)}{{\Phi }^{m+1}(r)}\right\}$$

In real calculations, the length N of the time series is a limited value. Therefore, sample entropy is typically expressed as:

21 $$SampleEn(m,r,N)=\mathrm{ln}\frac{{\Phi }^{m}(r)}{{\Phi }^{m+1}(r)}$$

SampleEn is not limited by the length of the time series, and the embedding dimension $m$ and allowable deviation $r$ contribute are the same for SampleEn. A higher SampleEn represents a dynamic system trend toward randomness with better adaptability. A lower SampleEn indicates a time series with higher periodicity. Meanwhile, the improved time series regularity algorithm of sample entropy is more suitable for the analysis of biological time series such as ECG^42^. Table 1 summarizes and describes the characteristic parameters used for further analysis in this study.

**Table 1:** Description of HRV parameters used in this study.

No	Parameters	Unit	Description
**Time domain parameters**			
1	meanRR	ms	The mean of RR intervals
2	meanHR	beats/min	The mean heart rate
3	SDNN	ms	Standard deviation of RR intervals
4	RMSSD	ms	Square root of the mean squared differences between successive RR intervals
5	SDSD	ms	Standard deviation of the difference between adjacent interval
6	CV	–	Ratio between SDNN and RR band powers
7	PNN20	%	NN20 divided by the total number of RR intervals
8	PNN50	%	NN50 divided by the total number of RR intervals
**Frequency domain parameters**			
1	VLF percent	%	Relative powers in very low frequency band (0–0.04 Hz)
2	LF percent	%	Relative powers low frequency band (0.04–0.15 Hz)
3	HF percent	%	Relative powers in high frequency band (0.15–0.4 Hz)
4	LF norm	–	Normalized low frequency power
5	HF norm	–	Normalized high frequency power
6	LF/HF	–	Ratio of LF power to HF power
**Nonlinear parameters**			
1	SD1	ms	Standard deviation for T direction of Poincare plot
2	SD2	ms	Standard deviation for L direction of Poincare plot
3	SD1/SD2	–	Ratio of SD1 to SD2
4	SampleEn	–	A method to measure the complexity of time series

## Analysis and results

**Figure 5:**
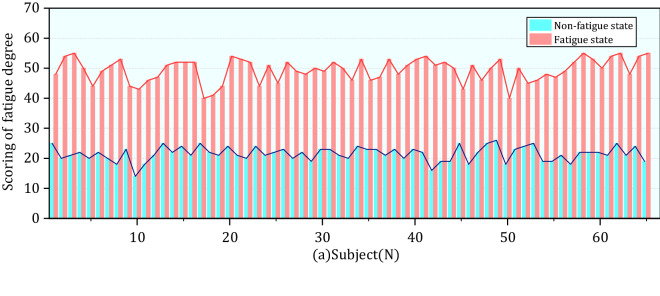
Total score of each subject in FS-14.

In this section, we calculated the characteristic values in the questionnaire and HRV for ECG signals and analyzed the correlation and change trend of each parameter. The results were given as the mean ± SD. After removing unavailable data due to weather and signal distortion, 65 out of 67 subjects were used for further analysis, including 38 males and 27 females. The state of fatigue was identified according to the scoring at the Fatigue Scale-14, the score of questionnaire FS-14 answered by orchard workers is shown in Table 2 and Fig. 5. Compared with before daily work, self-reporting total scores of orchard workers as daily work increased from 21.5 to 49.4 and the increase was significant (P < 0.001), we could conclude that as completing the day's work the orchards workers were in a state of fatigue.

**Table 2:** Characteristic values of Fatigue Scale-14.

State	Min–max	Mean ± SD 95%CI	Sig
Before daily work	14–26	21.5 ± 2.4	0.000**
After daily work	40–55	49.4 ± 3.9

Table 3 shows that the mean self-reported FS-14 broke into its subtraits of mental and physical fatigue of workers. The scoring of each section both increased significantly (P < 0.001), and the scoring of physical fatigue and mental fatigue on average increased 21.6 and 6.2, respectively. From the results of the questionnaire, it is clearly seen that both physiological and mental scores increased, but the increase in the physiological fatigue score was more compared to the mental fatigue score. Therefore, it can be inferred that physiological fatigue contributes more to the comprehensive fatigue of orchard workers. In this study, all subjects worked for about 6 h to complete daily tasks, but the content of work was not recorded for each subject. Therefore, in order to delay appearance of fatigue, it can be considered in terms of reducing the working hours and the work intensity.

**Table 3:** Characteristic values of physical and mental parts on the Fatigue Scale-14.

Score	Nonfatigue state 95%CI	Fatigue state 95%CI	Sig
Total score	21.5 ± 2.4	49.4 ± 3.9	0.000**
Physical score	12.6 ± 1.9	34.2 ± 3.4	0.000**
Mental score	9.0 ± 1.6	15.2 ± 1.8	0.000**

**Figure 6:**
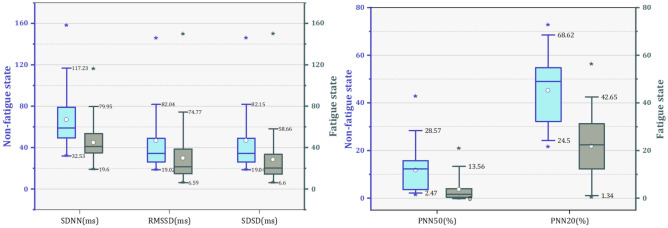
The value of SDNN, RMSSD, SDSD, PNN50 and PNN20 changes in the nonfatigue and fatigue state.

**Figure 7:**
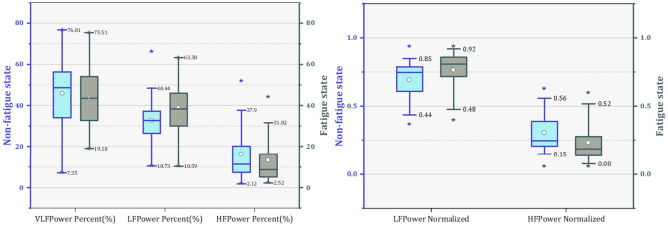
The value of VLF (%), LF (%), HF (%), LFnorm and HFnorm changes in the nonfatigue and fatigue state.

Next, the results of HRV parameters of subjects before and after fatigue are shown in this section. It was found that each parameter has different variation patterns. Table 4 shows the characteristic value changes of the HRV parameters of all subjects in the nonfatigue and fatigue states. Correlation coefficients were used to analyze the correlation of individual parameters, but the majority of HRV parameters did not pass the K-S normality test. Therefore, Spearman coefficients were chosen for analyze the specific relevance of each parameter and significant differences were tested by Mann–Whitney U test. The significant difference is expressed by the symbol “*”, “*” represents the significance level P < 0.05, and “**” represents the significance level P < 0.001. The results show that RMSSD (Spearman coefficient = 0.312), SDSD (Spearman coefficient = 0.389), LF norm (Spearman coefficient = 0.355), HFnorm (Spearman coefficient = 0.355), SD1 (Spearman coefficient = 0.375) SampleEn_iHR (Spearman coefficient = 0.453) and SampleEn_Peak (Spearman coefficient = 0.354) with the moderate correlations (Spearman coefficient > 0.3) in fatigue and nonfatigue states among time domain, frequency domain and nonlinear parameters. The strongest significant (P < 0.001) increased difference was found for mean HR LF/HF SampleiHR, followed by LF percent and Samplepeak, SampleRR. On the other hand, fatigue also led to significant decreases (P < 0.05) in nine HRV parameters (meanRR, SDNN, RMSSD, SDSD, PNN50, PNN20, LFnorm, SD1, SD2, SD1/SD2), of which the eight parameters had the stronger significance level (P < 0.001) for the decrease, and SD1/SD2 showed a decrease significanly (P < 0.05). Figures 6, 7 shows the differences of HRV parameters in nonfatigue and fatigue states.

**Table 4:** Characteristic values of each HRV parameter.

No	Parameter	Mean [SD]		Spearman	M-W U test
		Nonfatigue state	Fatigue state		
**Time domian**					
1	MeanHR	72.154 ± 8.987	86.692 ± 7.709	0.25258	**
2	MeanRR	844.971 ± 105.832	697.508 ± 66.827	0.24382	**
3	SDNN	67.122 ± 25.272	44.996 ± 17.444	0.25296	**
4	RMSSD	46.867 ± 34.475	29.938 ± 23.165	0.33115	**
5	SDSD	46.996 ± 34.589	28.615 ± 22.823	0.38888	**
6	PNN50	11.85 ± 8.917	3.742 ± 4.678	0.22654	**
7	PNN20	45.316 ± 14.108	21.915 ± 13.115	0.22888	**
8	CV	0.082 ± 0.032	0.064 ± 0.023	0.31081	**
**Frequency domain**					
1	VLF percent	46.024 ± 15.71	43.535 ± 14.379	0.13783	NS
2	LF percent	32.831 ± 10.199	38.927 ± 12.21	0.03427	*
3	HF percent	16.36 ± 12.209	13.444 ± 11.171	0.2691	NS
4	LF norm	0.694 ± 0.134	0.766 ± 0.14	0.35475	NS
5	HF norm	0.306 ± 0.134	0.234 ± 0.14	0.35475	**
6	LF/HF	2.976 ± 2.058	4.842 ± 3.268	0.35082	**
**Nonlinear**					
1	SD1	33.231 ± 24.458	20.214 ± 14.24	0.37514	**
2	SD2	86.193 ± 31.777	59.97 ± 21.213	0.20016	**
3	SD1/SD2	0.38 ± 0.205	0.326 ± 0.177	0.2353	*
4	SampleEn_Peak	1.522	1.7475	0.354	*
5	SampleEn_RR	1.2565	1.589	0.152	*
6	SampleEn_iHR	1.2165	1.342	0.453	**

In order to determine the differences in the variation of HRV parameters in different groups, we grouped all subjects by gender and discussed the change of HRV parameters in different genders separately. The differences in HRV between fatigue and nonfatigue states for orchard workers of different genders can be seen in the following Table 5.

**Table 5:** Characteristic values of time domain parameters in HRV.

No	Parameter	Mean [SD]				M-W U test	
		M-Nonfatigue	M-Fatigue	F-Nonfatigue	F-Fatigue	MN-MF	FN-FF
**Time domian**							
1	MeanHR	73.658 ± 8.666	84.947 ± 8.466	70.037 ± 9.163	89.148 ± 5.796	**	**
2	MeanRR	825.791 ± 98.837	713.445 ± 75.162	871.966 ± 111.238	675.08 ± 45.387	**	**
3	SDNN	66.814 ± 23.07	51.137 ± 19.351	67.554 ± 28.537	36.353 ± 9.133	*	**
4	RMSSD	54.139 ± 41.559	36.927 ± 27.435	36.632 ± 16.783	20.103 ± 8.829	NS	**
5	SDSD	54.209 ± 41.614	34.639 ± 27.501	36.844 ± 17.248	20.137 ± 8.846	*	**
6	PNN50	10.43 ± 7.994	4.264 ± 5.004	13.849 ± 9.882	3.007 ± 4.155	**	**
7	PNN20	43.491 ± 14.486	23.95 ± 14.184	47.885 ± 13.402	19.051 ± 11.069	**	**
8	CV	0.085 ± 0.033	0.071 ± 0.027	0.077 ± 0.03	0.053 ± 0.012	NS	**

Eight of the time-domain HRV parameters were analyzed. Due to the alteration in cardiac rhythm under fatigue, the related characteristic parameters displayed certain trends. In male orchard workers, four time-domain parameters showed significant changes (P < 0.05), among which meanHR increased significantly and meanRR, SDSD, PNN50, PNN20 decreased significantly. However, as for female orchard workers, all 8 HRV parameters showed significant changes (P < 0.05), only meanHR showed an increasing trend, and the remaining time-domain parameters RR SDNN RMSSD, SDSD, PNN50, PNN20, CV showed significant decreases (P < 0.05).

**Figure 8:**
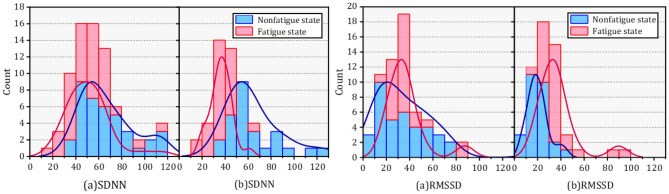
The probability density function of SDNN and RMSSD in different gender under nonfatigue and fatigue state.

**Figure 9:**
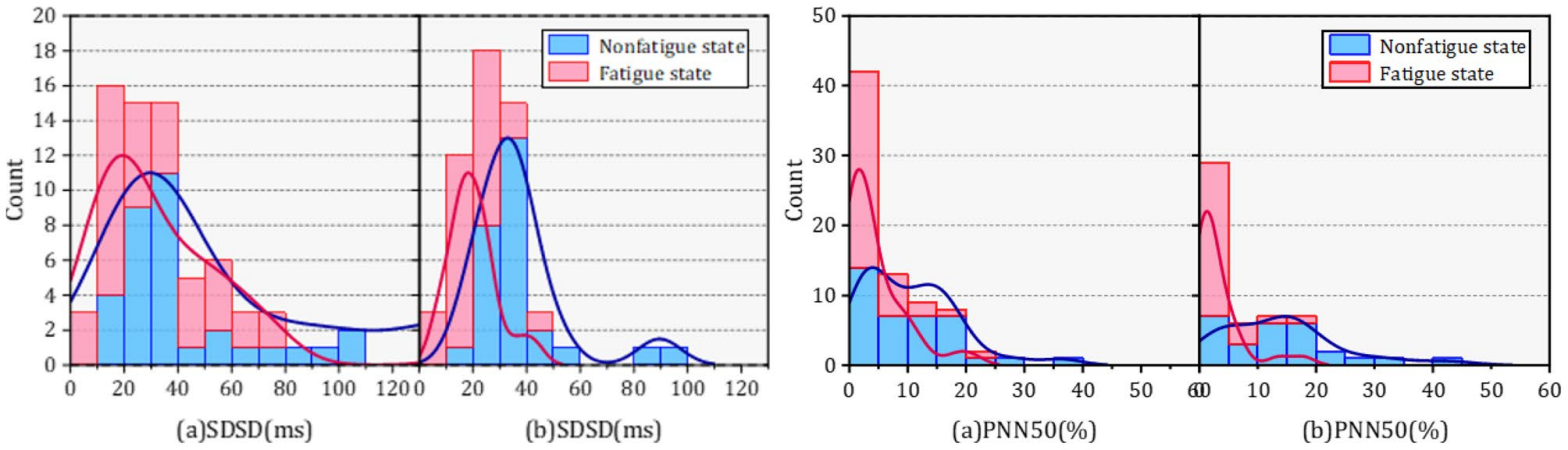
The probability density function of SDNN and RMSSD in different gender under nonfatigue and fatigue state.

**Figure 10:**
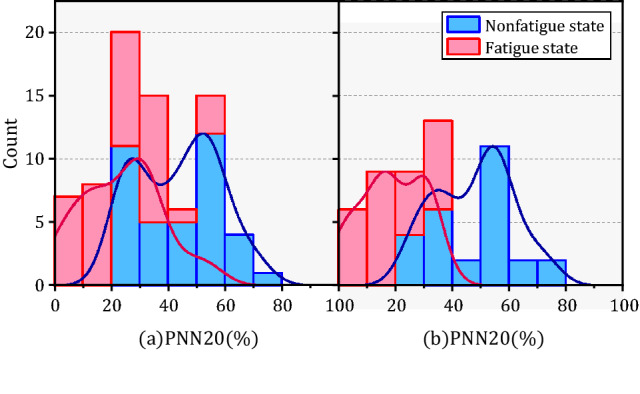
The probability density function of SDNN and RMSSD in different gender under nonfatigue and fatigue state.

The probability density functions (PDF) of HRV parameters for orchard workers of different genders also showed distinct differences as seen in Figs. 8, 9, 10, (a) represents male and (b) represents females. The distribution of SDNN and SDSD in fatigue and nonfatigue states in the time domain parameters was obviously different among the different gender of orchard workers. The peak segment of the PDF of SDNN for female workers increased significantly after fatigue, but the number remained almost constant in males. In addition, although the SDNN shifted to the lower segment after fatigue, the number of SDNN distributed in the peak segment increased in male workers, but decreased in females compared to the nonfatigue condition. The PDF of the remaining time-domain parameters did not change significantly in the gender differences.

In the frequency domain, six parameters were analyzed, and it was found that each parameter was significantly different. Table 6 presents the results of the parameters in the frequency domain before and after the task.

**Table 6:** Characteristic values of frequency domain parameters in HRV.

No	Parameter	Mean [SD]				M-W U test	
		M-Nonfatigue	M-Fatigue	F-Nonfatigue	F-Fatigue	MN-MF	FN-FF
**Frequency domain**							
1	VLF percent	43.282 ± 15.823	44.91 ± 15.319	49.883 ± 14.993	41.6 ± 12.973	NS	*
2	LF percent	32.824 ± 8.618	36.716 ± 11.146	32.841 ± 12.262	42.038 ± 13.152	NS	*
3	HF percent	19.488 ± 13.534	14.404 ± 11.703	11.957 ± 8.472	12.094 ± 10.442	*	NS
4	LF norm	0.659 ± 0.147	0.752 ± 0.138	0.744 ± 0.097	0.786 ± 0.143	*	*
5	HF norm	0.341 ± 0.147	0.248 ± 0.138	0.256 ± 0.097	0.214 ± 0.143	*	*
6	LF/HF	2.515 ± 1.518	4.482 ± 3.202	3.626 ± 2.53	5.349 ± 3.352	*	*

The relative power of three different frequency bands showed distinct changes in nonfatigue and fatigue. First, among male orchard workers, only HFpercent showed a significant decrease, while VLFpercent and LFpercent showed a trend of change but did not produce a significant difference. In contrast, a significant upward trend in both VLFpercent and LFpercent was observed in female orchard workers. The normalized method was applied to process frequency domain parameters (LF and HF) to ensure the stability and accuracy of the data features. Furthermore, a better uniformity and significant differences in the three normalized parameters were obtained. Both LFnorm and HFnorm showed significant increases and decreases in male and female workers, respectively. Meanwhile, the ratio of LF to HF also showed a significant increase compared to nonfatigue state of workers.

**Figure 11:**
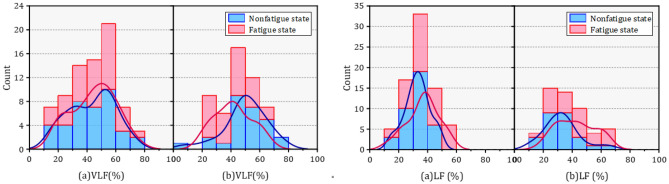
The probability density function of VLF (%) and LF (%) in different gender under nonfatigue and fatigue state.

**Figure 12:**
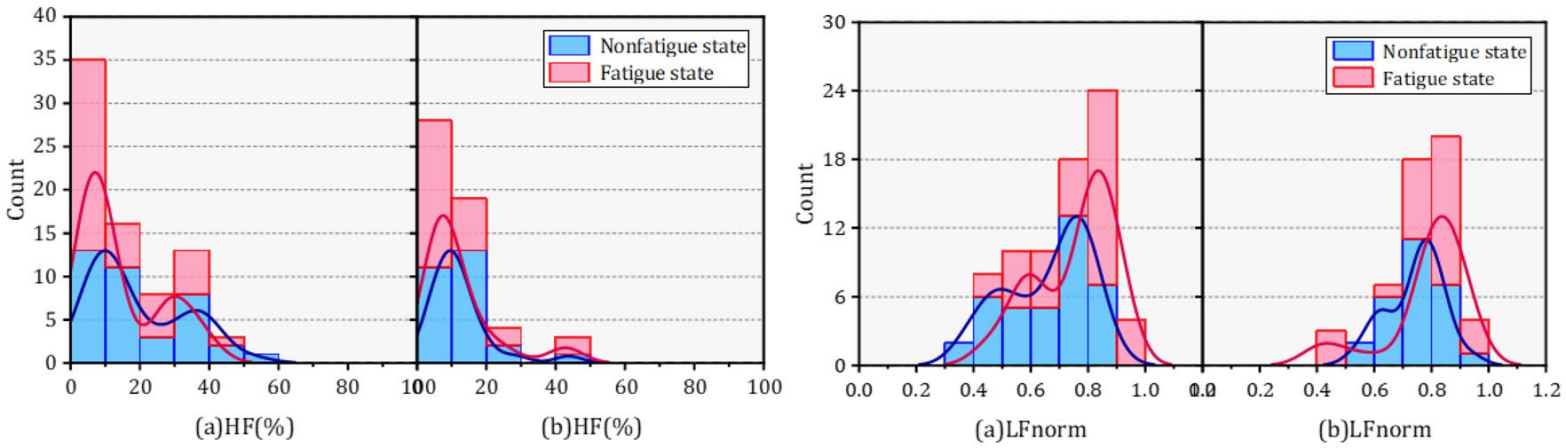
The probability density function of HF (%) and LFnorm in different gender under nonfatigue and fatigue state.

**Figure 13:**
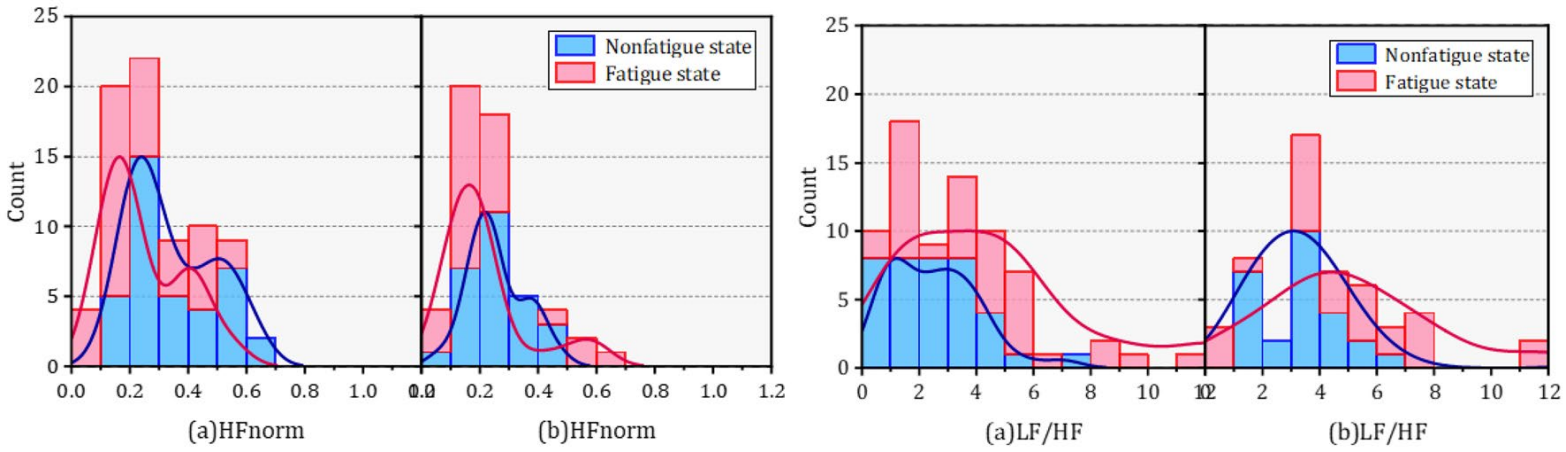
The probability density function of HFnorm and LF/HF in different gender under nonfatigue and fatigue state.

The variation of the PDF of each frequency domain parameter is shown in Figs. 11, 12, 13. The VLF percent distribution in the peak PDF segment showed a slight increase after fatigue in male workers, but this decreased in females. In addition, the PDF of HFnorm peak segment of remained unchanged before and after fatigue in male workers, but there has been a significant decline among women. The PDF of LF/HF in fatigue and nonfatigue states were almost opposite in different genders.

Changes in VLF percent were considered to originate from thermal and hormonal control along with vasomotor activity. LFnorm and HFnorm were supposed to reflect cardiac sympathetic activity and vagal activity, respectively. Likewise, the LF/HF ratio was used to estimate the balance between cardiac sympathetic and parasympathetic activity.

In nonlinear analysis, six nonlinear parameters of HRV parameters were calculated and summarized in Table 7. SD1 and SD2 of the Poincaré plot generally measure short-term and long-term variability features in ms.

**Table 7:** Characteristic values of nonlinear parameters.

No	Parameter	Mean [SD]				M-W U test	
		M-Nonfatigue	M-Fatigue	F-Nonfatigue	F-Fatigue	MN-MF	FN-FF
**Nonlinear**							
1	SD1	38.331 ± 29.426	24.46 ± 16.694	26.053 ± 12.196	14.239 ± 6.255	*	**
2	SD2	82.434 ± 24.778	67.816 ± 22.419	91.484 ± 39.525	48.927 ± 13.258	*	**
3	SD1/SD2	0.44 ± 0.239	0.34 ± 0.177	0.296 ± 0.096	0.306 ± 0.178	*	NS
4	SampleEn_Peak	1.605 ± 0.274	1.812 ± 0.382	1.439 ± 0.272	1.683 ± 0.3431		**
5	SampleEn_RR	1.401 ± 0.2629	1.996 ± 0.2535	1.112 ± 0.19	1.182 ± 0.35	*	*
6	SampleEn_iHR	1.244 ± 0.497	1.37 ± 0.616	1.189 ± 0.316	1.314 ± 0.283	*	**

In this study, SD1 and SD2 showed a significant decrease (P < 0.05) in both male and female workers. And among male orchard workers, The SD1 decrease was less than SD1, leading to a smaller SD1/SD2 and indicating the shift of balance between short-term variability and long- term variability in the RR interval. but SD1/SD2 did not show a significant change (P > 0.05) among female workers.

**Figure 14:**
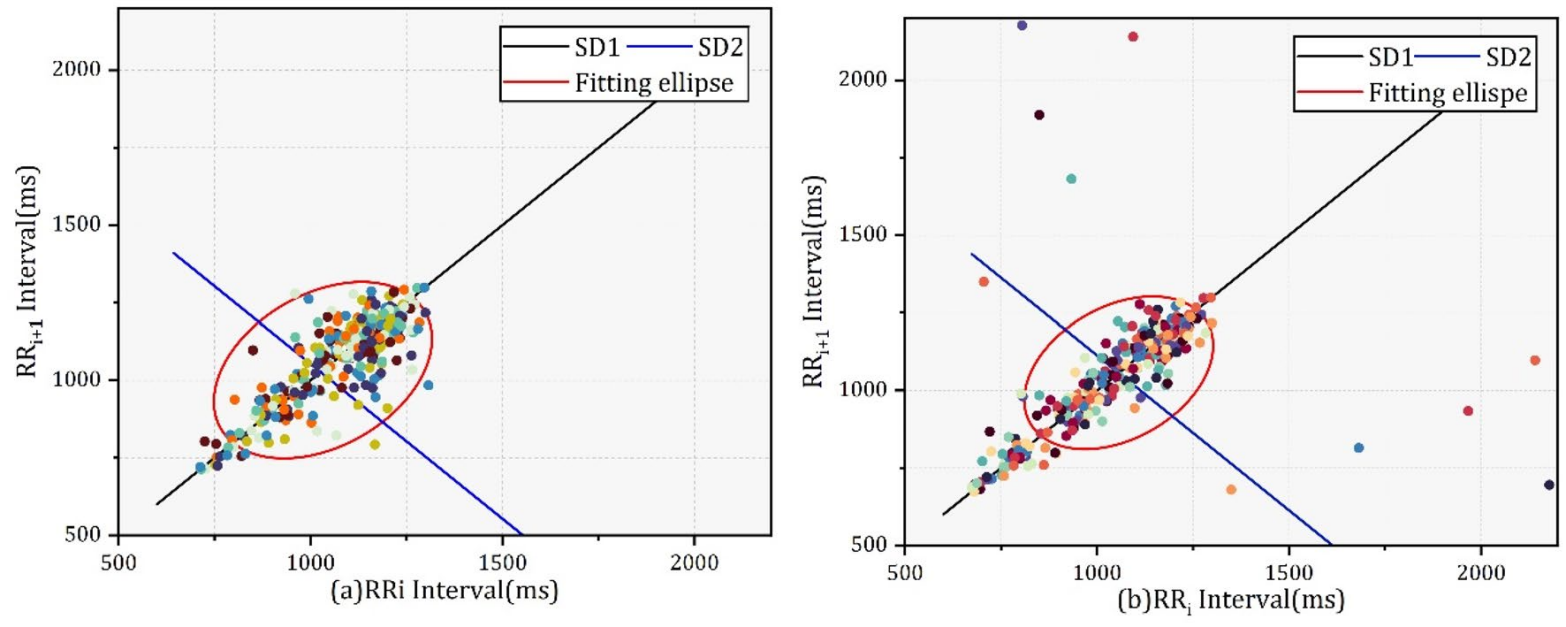
Poincare plot of orchard workers in the nonfatigue state (**a**) and fatigue state (**b**).

The Poincaré plot of a normal subject usually has a comet shape, and data points distributed in the head of the scatter graph are dense and relatively loose in the end. In this study, Fig. 14a,b shows the Poincaré plot of orchard workers in fatigue and nonfatigue states. The distribution area of RR intervals in the Poincaré plot decreased than that in the nonfatigue state, and several outliers appeared at the edge of the Poincaré plot, which represented that the RR intervals of subjects after the operating task became relatively unstable.

**Figure 15:**
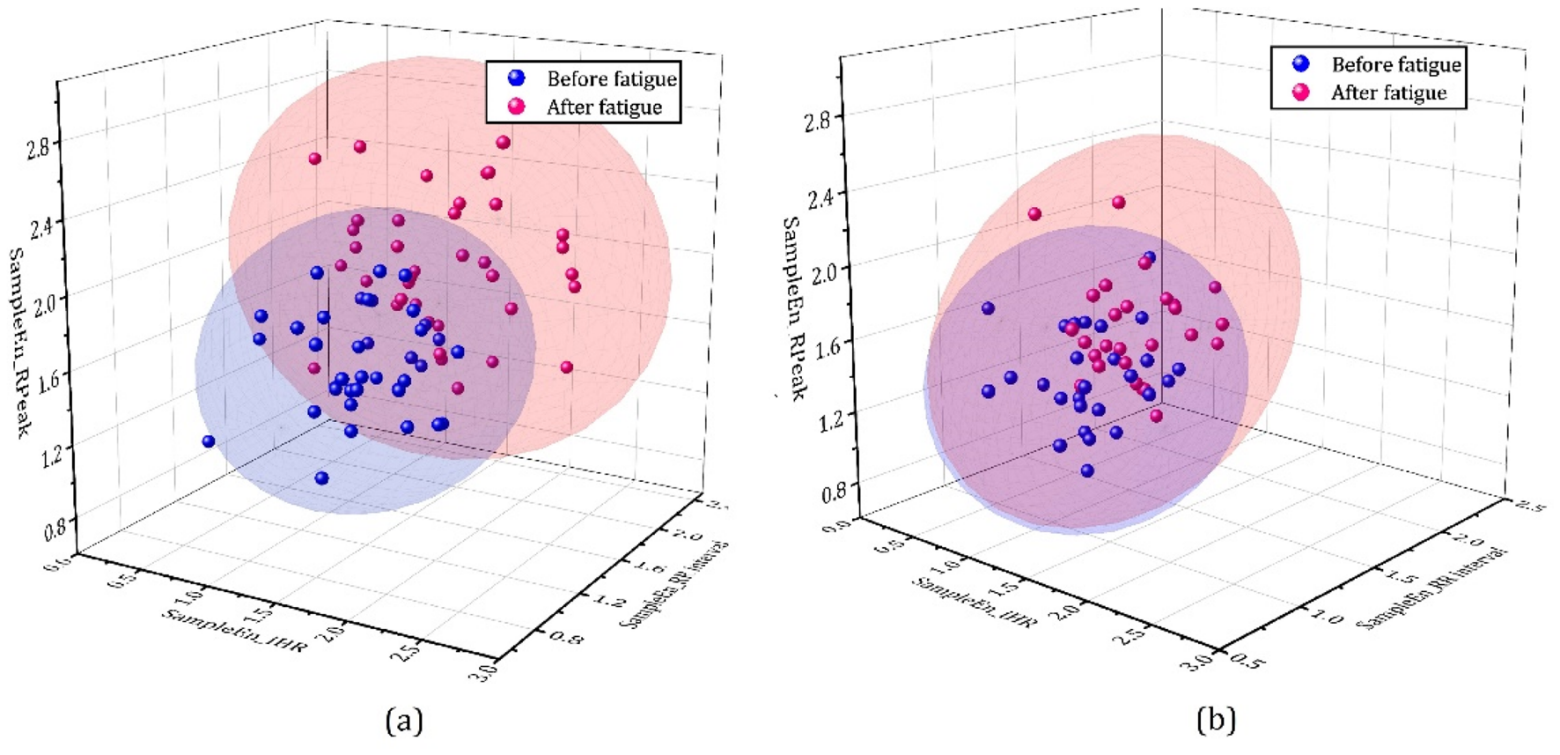
The result sample entropy analysis of iHR, RR interval and value of R Peak in different gender of workers.

The sample entropy of instantaneous heart rate (SampleEn_iHR), RR interval (SampleEn_RR) and value of R wave peak (SampleEn_Peak) was calculated for analyzing complexity differences of ECG signal. It is can be seen from the Fig. 15 that the characteristic value of the sample entropy analysis become more discrete than nonfatigue state. Specifically, SampleEn_Peak SampleEn_iHR SampleEn_RR showed an increasing trend among male orchard workers relative to the nonfatigued state, but no significant difference was observed in the change of SampleEn_Peak. The difference was that SampleEn_iHR had the strongest significant level among female orchard workers.

Both SampleEn_iHR, SampleEn_RR and SampleEn_Peak were positive numbers, which indicates the dynamic performance of the ECG signal in the chaotic state. Under the fatigue state, the unpredictability and complexity increased in SampleEn_iHR and SampleEn_Peak, while this decreased in SampleEn_RR. Thus, the confusion of instantaneous heart rate and value of R wave peak increased and RR interval decreased in fatigue state than nonfatigue state. The results of nonlinear analysis can provide valid information on occupational health disease diagnosis.

## Discussion

This study analyzed the nonfatigue and fatigue state of orchardworkers with a change in the HRV of ECG signals caused by daily work under hilly orchard conditions. According to field data collection and further analysis, we found the HRV difference of orchard workers between nonfatigue state and fatigue these differences were observed both in time domain, frequency domain and nonlinear parameters.

We summarized the change trend of each parameter mentioned above, which can be seen in Table 8. The seven time-domain parameters, including MeanRR (decreased − 39.11%), PNN50 (decreased 68.42%), PNN20 (decreased 51.64%) and CV (decreased 21.95%) were significantly smaller in the fatigue state than in the nonfatigue state. Conversely, the MeanHR (increased 20.15%) was larger in the fatigue state. The changes of time domain parameters could be explained by limb movement activated by the reciprocal activation pattern of the body, and autonomic nerves play an increasingly dominant role in the regulation of cardiac rhythm. Moreover, SDSD showed a different distribution in PDF among different gender of orchard workers. In frequency domain, one of the relative power parameters LF percent (increased 17.82%) was larger than the nonfatigue state, and the other two parameters (VLF percent decreased 5.41% and HF percent decreased 17.82%) showed a decreasing trend. However, the significance of three relative power parameters varies greatly among different genders and the PDF of VLF percent, HF norm and LF/HF in fatigue and nonfatigue state differed significantly. The lower HF percent and HFnorm indicated that the regulation of vagus nerve activity was inhibited in the fatigue state. Correspondingly, the simultaneous increase in LFnorm means that the dual regulation of sympathetic and parasympathetic nerves to the cardiac rhythm becomes more sensitive^43,44^. The marked increase in the LF/HF ratio (increased62.7%) can be explained by the gap in the activation level of the cardiac sympathetic and parasympathetic systems becoming wider with fatigue than without fatigue.

**Table 8:** The changing trend of each parameter used in this study.

No	Parameter	Unit	State		Rate (%)
			Non fatigue	Fatigue	
1	MeanRR	ms	844.971	697.499	− 17.45%
2	MeanHR	beats/min	72.154	86.692	20.15
3	SDNN	ms	67.122	44.996	− 32.96
4	RMSSD	ms	46.867	29.938	− 36.12
5	SDSD	ms	46.996	28.615	− 39.11
6	PNN50	%	11.85	3.742	− 68.42
7	PNN20	%	45.316	21.915	− 51.64
8	CV	–	0.082	0.064	− 21.95
9	VLF percent	%	46.024	43.535	− 5.41
10	LF percent	%	32.831	38.927	18.57
11	HF percent	%	16.36	13.444	− 17.82
12	LFnormalized	–	0.694	0.766	10.37
13	HFnormalized	–	0.306	0.234	− 23.53
14	LF/HF	–	2.976	4.842	62.7
15	SD1	ms	33.231	20.214	− 39.17
16	SD2	ms	86.193	59.97	− 30.42
17	SD1/SD2	–	0.38	0.326	− 14.21
18	SamEntropy_iHR		1.2165	1.342	10.316
19	SamEntropy_RR		1.2565	1.589	26.46
20	SamEntropy_Peak		1.522	1.7475	14.81

Three parameters, SD1 (decreased 39.17%), SD2 (decreased 30.42%) and SD1/SD2 (decreased 14.21%), in the Poincaré plot for nonlinear analysis showed decreasing trends. However, there was no significant difference change in SD1/SD2 among female orchard workers when gender differences were considered. Poincare plot analysis is an emerging quantitative-visual technique whereby the shape of the plot is categorized into functional classes that indicate the degree of the abnormal heart rate in a subject and provides summary information as well as detailed beat-to-beat information on the behavior of the heart^45,46^. As shown in Fig. 14, the differences between the two figures described the abnormal change in heart rate caused by an imbalanced RR interval in the fatigue state. In the analysis of sample entropy, Three characteristic value showed a slight increase, but there was no significant difference in the SampleEn_Peak of male orchard workers in fatigue and nonfatigue state. The result of nonlinear analysis indicates that the degree of disorder and complexity in cardiac rhythm increased, which means that the heart rhythm regulation ability of hilly orchard workers is limited due to fatigue.

Lu et al.^47^ proposed a facial recognition method for tractor driver fatigue based on a convolutional neural network. After gamma brightness correction and wavelet denoising for the collected image, the PCA-SCM core feature recognition algorithm was used to identify the driver face. Through the comparison of four detection methods in field tests, including the back propagation neural network, dynamic template matching technique, fuzzy reasoning method and convolutional neural network, the convolution neural network achieved the best recognition rate and reached 98.9%. Chen et al.^48^ used linear and nonlinear methods to analyze the difference in miners' HRV before and after fatigue. The results show that LFnorm, HFnorm and the HF/LF ratio exhibited increasing trends and significant differences. It is suggested that SDNN, LF, HF, LF/HF, HR, RH and RR are gold-standard sensitive parameters that can be used to reliably detect miner fatigue. Huang et al.^49^ used wearable ECG smart devices to detect mental fatigue. They found that the rMSSD was positively associated with the mental fatigue state and that the PNN50 and NN.mean were negatively associated with the mental fatigue state. Moreover, this study also combined different indicators in four classification algorithms. It was found that NN.mean, TP and LF achieved the best accuracy, which was 75.5% with KNN (k = 3), and NN.mean, PNN50, TP and LF were the key HRV indicators identified for mental fatigue detection. Tong et al.^50^ tested the relationship between the HRV parameters and exercise-induced fatigue and found that the SDNN, RMSSD, and PNN50 of nine long-term runners decreased after exercise. Likewise, it is generally believed that drowsiness is also a manifestation of fatigue. However, some studies showed opposite results under drowsy conditions. Byeon et al.^51^ and Patel et al.^52^ studied driving fatigue in ECG signals, and a marked decrease was shown in the LF to HF ratio in drivers’ fatigue state, while other studies^4,53^ showed that LF/HF may increase with higher fatigue levels during tasks. Therefore, the results of these studies are still in dispute.

Liu et al.^54^ extracted the RR interval series and calculated the value of approximate entropy (An) in normal sinus rhythm (NSR), ventricular tachycardia (VT) and ventricular fibrillation. The results show that the ApEn increased significantly from NSR to VT and then to VF, which may be regarded as an index to discriminate the ECG signals of different states. Zhang et al.^55^ and Chen et al.^48^ analyzed driving fatigue and miners' working fatigue by sample entropy. The former study extracted the wavelet entropy (WE), the peak-to-peak value of the approximate entropy (PP-ApEn) and sample entropy (PP-SampEn) in real-time ECG signals, and an artificial neural network (ANN) model was applied to recognize the fatigue state of drivers. The automatic identification of driver fatigue was achieved, and the accuracy of estimation was approximately 96.5–99.5%. The result of the miner fatigue state study indicates that the SampleEn of the RR interval and HR increased from the nonfatigue state to the fatigue state. The results in this study are line with these studies.

## Conclusion

In this study, HRV and sample entropy analyses of ECG signals were used to research the differences in the nonfatigue and fatigue states of orchard workers. Sixty-five healthy orchard workers (38 men and 27 women) without cardiovascular disease were recruited from hilly orchards in South China. The fatigue state was identified by a subjective questionnaire (FS-14), and sixteen linear HRV parameters and six nonlinear parameters in short-term intervals (5 min) were calculated before and after the experiment. After this, Spearman correlation analysis and M-W Utests were performed on each parameter, and convincing results were obtained, which can be summarized as follows:

1. The fatigue scale questionnaire scoring increased 27.9 on average from a nonfatigue state to a fatigue state. In particular, the degree of physical fatigue and mental fatigue of orchard workers all had upward trends, which increased on average 21.6 and 6.2 on average in scoring, respectively. The physiological fatigue contributed more to the comprehensive fatigue of orchard workers It can be considered to delay the occurrence of fatigue by reducing work intensity and working hours
2. Among all HRV parameters, RMSSD, SDSD, CV, LFnorm, HFnorm, LF/HF, SD1, SampleEn_Peak and SampleEn_iHR with Spearman coefficients larger than 0.3, however, the remaining HRV parameters had correlation coefficients less than 0.3 with almost no correlation. Without considering the effect of gender, 6 HRV parameters (meanHR, LFpercent, LF/HF, SampleEn_iHR, SampleEn_RPeak, SampleEn_RR) showed a significant increase (P < 0.05) and 10 HRV parameters (meanRR, SDNN, RMSSD, SDSD, PNN50, PNN20, CV, HFnorm, SD1, SD2 and SD1/SD2) showed a significant decrease (P < 0.05), which results suggest that these HRV characteristics can be used to distinguish the ability of orchard workers' fatigue status.
3. Fatigue led to significant changes in 15 HRV parameters among male hilly orchard workers, but in females 18 HRV parameters showed significant changes. The nonlinear parameters all showed a tendency to disperse in the fatigue state. In addition, the PDF of SDNN, SDSD, VLF%, HFnorm and LF/HF were significantly different in the fatigue and nonfatigue states of the different genders of orchard workers, which leads to the conclusion that there are gender differences in the effects of fatigue on the autonomic nervous system. It can be inferred that more accurate results can be obtained when the HRV parameter is used to distinguish the fatigue status of different genders of orchard workers.

There are several limitations in this study. First, we collected ECG samples from 67 orchard workers, and only 65 subjects were used for further analysis in this study. The small sample size is an inevitable factor in similar works. Second, due to the working conditions and environment in hilly orchards, we could not collect the real-time ECG signals of workers. Third, the experiment was conducted in a typical hilly orchard in southern China. Limited by regional factors, there are few studies in this field. Therefore, in future studies, more samples should be obtained to improve reliability, and the regional limitations of this study can be reduced by selecting more different hilly orchards for experiments. At the same time, the results of this study can be used to identify the fatigue state of orchard workers and provide a reliable reference for clinical medical diagnosis of cardiovascular disease.
